# Bioactive Compounds with Leishmanicidal Potential from *Helianthus tuberosus* and *Vernonanthura squamulosa*

**DOI:** 10.3390/molecules30051039

**Published:** 2025-02-24

**Authors:** Rachel Nápoles Rodríguez, María Laura Arreguez, Aldana M. Corlatti, Hernán G. Bach, César A. N. Catalán, Laura C. Laurella, Paola A. Barroso, Valeria P. Sülsen

**Affiliations:** 1 CONICET-Universidad de Buenos Aires, Instituto de Química y Metabolismo del Fármaco (IQUIMEFA), Autonomous City of Buenos Aires C1113AAD, Argentina; rnapolesrodriguez@gmail.com (R.N.R.); aldanamalencorlatti@gmail.com (A.M.C.);; 2 CONICET-Universidad Nacional de Salta, Instituto de Patología Experimental (IPE), Salta A4400, Argentina; arreguezmarialaura@yahoo.com.ar; 3 Universidad de Buenos Aires, Facultad de Farmacia y Bioquímica, Cátedra de Farmacognosia, Autonomous City of Buenos Aires C1113AAD, Argentina; 4 Instituto Nacional de Tecnología Agropecuaria, Buenos Aires B1686, Argentina; bach.hernan@inta.gob.ar; 5 Instituto de Química Orgánica, Facultad de Bioquímica, Química y Farmacia, Universidad Nacional de Tucumán, San Miguel de Tucumán, Tucumán T4000INI, Argentina; cancatalan@gmail.com

**Keywords:** Asteraceae, sesquiterpene lactones, *Leishmania amazonensis*, in vitro and in vivo assays

## Abstract

Leishmaniasis is a neglected tropical disease caused by protozoan parasites of the genus *Leishmania*. An estimated 700,000 to 1 million new cases occur annually. Current therapies are limited by high toxicity, cost, prolonged treatment period, and rising resistance in endemic regions. The Asteraceae family has emerged as a promising source of bioactive compounds with proven leishmanicidal activity. In this study, the assessment of the antileishmanial activity of *Helianthus tuberosus* and *Vernonanthura squamulosa* extracts, the isolation of the sesquiterpene lactones heliangin and glaucolide A, respectively, and the evaluation of the activity of the compounds were conducted. Dichloromethane extracts of *H. tuberosus* and *V. squamulosa* were active on *Leishmania amazonensis* promastigotes, inhibiting the replication of the parasites in 97.2 ± 3.1% and 89.1 ± 1.1%, respectively, at 100 μg/mL. Heliangin was active against promastigotes of *L. amazonensis* (IC_50_ = 9.3 μM) and intracellular amastigotes (IC_50_ = 0.8 μM), while glaucolide A exhibited moderate activity against promastigotes (IC_50_ = 46.7 μM) and did not show activity against intracellular amastigotes. Based on these results, heliangin was further evaluated in an animal model of cutaneous leishmaniasis using BALB/c mice infected with *L. amazonensis*. Heliangin (8 mg/Kg), when administered in combination with Glucantime, significantly reduced lesion progression and parasite load compared to the vehicle-treated group (*p* < 0.001). These findings show that heliangin is a potential candidate for leishmaniasis treatment, especially in combination with therapeutic drugs.

## 1. Introduction

Neglected tropical diseases (NTDs) are a group of infectious diseases that primarily affect marginalized populations living in low-resource settings, particularly in tropical and subtropical regions. These diseases often have devastating health, social, and economic impacts, with significant morbidity and mortality affecting more than one billion people worldwide and contributing to an estimated 200,000 deaths and 19 million disability-adjusted life years lost annually [1].

Leishmaniasis is a parasitic NTD caused by protozoa of the genus *Leishmania* and transmitted by the bite of infected female phlebotomine sandflies. There are more than 20 *Leishmania* species that can infect humans, leading to different clinical manifestations of the disease. The three main forms are visceral, cutaneous, and mucocutaneous leishmaniasis, depending on the *Leishmania* species involved. Cutaneous leishmaniasis is the most common form, presenting as skin lesions, while visceral leishmaniasis is the most severe, and is potentially life-threatening if left untreated [2]. Leishmaniasis remains a significant public health issue in four major geographic regions globally: the Americas, East Africa, North Africa, and Western and Southeastern Asia [3]. An estimated 700,000 to 1 million new cases occur annually [2]. The first line of treatment for leishmaniasis is chemotherapy. Antimonials are the first therapy option, followed by miltefosine, amphotericin B, paromomycin and different combination therapies. These drugs have limited safety and efficacy and several disadvantages such as high costs, considerable toxicity, and the emergence of drug-resistant parasites that limit their use [4,5]. Hence, the search for novel treatment alternatives for this parasitic disease is crucial.

Natural products have been a valuable source of active compounds that play a crucial role in the discovery and development of new therapeutic agents [6]. Notable examples, in the area of antiparasitic drugs, include quinine and artemisinin, which are plant-derived compounds used as antimalarials, along with their derivatives [7]. The Asteraceae family includes a diverse array of plant species. Many members of this family possess therapeutic properties and have the potential to contribute to the development of novel medicinal treatments. The plants of this family produce a variety of secondary metabolites, such as terpenoids and flavonoids which hold promise for the development of new drugs to treat neglected protozoan diseases [8]. *Helianthus tuberosus* L. and *Vernonanthura squamulosa* (Hook. & Arn.) H. Rob. are two Asteraceae medicinal species. *H. tuberosus*, known as Jerusalem artichoke or Topinambur, has been used to stimulate the immune system and for wound healing, among other uses [9]. *Vernonanthura* species have been used to treat cough, bronchitis, flu, colds, bleeding, uterine infections and for other ailments [10]. Sesquiterpene lactones of the heliangolide and glaucolide types have been isolated from these species as well as from other Asteraceae and some of them have shown antiparasitic activity [11,12].

Other plant-derived secondary metabolites are being extensively investigated for their anti-leishmanial potential. One prominent group is diarylheptanoids, complex phenolic compounds known for their anti-parasitic, anti-inflammatory, and other beneficial properties. The synthesis of this type of compound has gained increasing attention in recent years due to their biological activities [13,14]. In this sense, the leishmanicidal activity of sixteen tetrahydropyran-based compounds, synthesized from centrolobine and diaspongins A and B, was evaluated. Of these, five compounds exhibited the strongest leishmanicidal effects while remaining non-toxic to human macrophages [15]. Additionally, des-O-methylcentrolobine, a phenolic diarylheptanoid from *Centrolobium sclerophyllum*, was found to be highly effective against *L. amazonensis* promastigotes. Curcumin, another well-known diarylheptanoid, has been extensively studied for its protective effects against various diseases [15]. Both curcumin and its derivatives have shown significant in vitro and in vivo anti-leishmanial activity against different Leishmania species [16].

Considering the necessity to find novel drugs to treat leishmaniasis, the medicinal properties of *H. tuberosus* and *V. squamulosa*, as well as the presence of sesquiterpene lactones on these species, the aim of this study was to evaluate the effect of crude extracts of both Asteraceae on *Leishmania amazonensis*, one of the species responsible for cutaneous leishmaniasis in Latin America, and to isolate the active compounds present in them.

## 2. Results

### 2.1. Leishmanicidal Activity of Dichloromethane Crude Extracts from Helianthus tuberosus and Vernonathura squamulosa

The crude dichloromethane extracts of *H. tuberosus* and *V. squamulosa* were assessed for their leishmanicidal activity against *L. amazonensis* promastigotes. The extract from *H. tuberosus* achieved a remarkable 97.2 ± 3.1% inhibition of parasites at a concentration of 100 μg/mL and 29.8 ± 8.5% inhibition at 10 μg/mL. In contrast, the extract from *V. squamulosa* showed leishmanicidal activity with inhibitions of 89.1 ± 1.1% and 9.17 ± 0.16% at 100 and 10 μg/mL, respectively.

### 2.2. Isolation and Identification of Compounds from Dichloromethane Crude Extracts of Helianthus tuberosus and Vernonanthura squamulosa

The fractionation of the active crude dichloromethane extract of *H. tuberosus* led to the isolation of an amorphous white precipitate, named compound A. This compound was obtained from fractions 27 to 32, yielding a total of 12 mg. The purity of compound A was 91.88% as assessed by HPLC (Figure S1).

**Figure 1:**
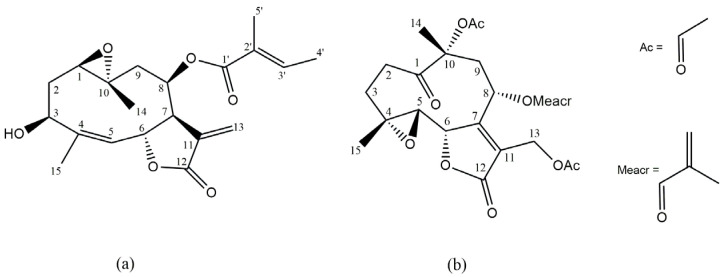
Chemical structure of the sesquiterpene lactones heliangin (**a**) and glaucolide A (**b**).

The structure of compound A was elucidated through spectroscopic analyses and by comparison with the literature [17]. Compound A was identified as the sesquiterpene lactone (STL) heliangin (Figure 1a).

On the other hand, compound B was isolated from the dewaxed dichloromethane extract of *V. squamulosa,* resulting in a total yield of 710 mg. The purity of this compound determined by HPLC was 96.53% (Figure S2). Compound B was identified by spectroscopic methods and by comparison with data in the literature [18] as the STL glaucolide A (Figure 1b). The NMR data of heliangin and glaucolide A are detailed in the Supplementary Materials (Tables S1 and S2).

### 2.3. In Vitro Leishmanicidal Activity of the Isolated Compounds

**Figure 2:**
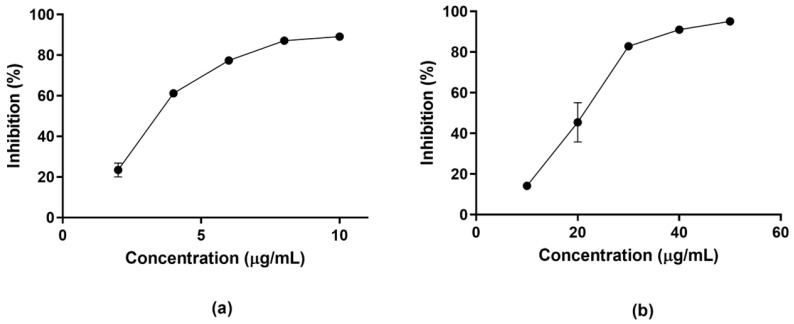
Leishmanicidal activity on *L. amazonensis* promastigotes of heliangin (**a**) and glaucolide A (**b**). Parasites were cultured for 48 h in the presence of the compounds. Parasite viability was assessed by measuring absorbance at 450 nm using a microplate reader (Tecan, Mannedorf, Switzerland). A dose–response sigmoidal curve was constructed to determine the median inhibitory concentrations (IC_50_) using GraphPad Prism 6 (GraphPad Software Inc., Dotmatics, Boston, MA, USA). The values represent the mean ± SD from three independent experiments conducted in triplicate.

The leishmanicidal activity of the STLs heliangin and glaucolide A was evaluated in vitro against *L. amazonensis*. The anti-leishmanial activity was first assessed on promastigotes. Heliangin demonstrated stronger activity with an IC_50_ value of 3.4 μg/mL (9.3 μM) (Figure 2a). In contrast, glaucolide A exhibited moderate activity against this parasitic form of *L. amazonensis*, with an IC_50_ of 21.7 μg/mL (46.7 μM) (Figure 2b). The reference drug (amphotericin B) showed an IC_50_ value of 0.1 μg/mL (Table 1).

**Figure 3:**
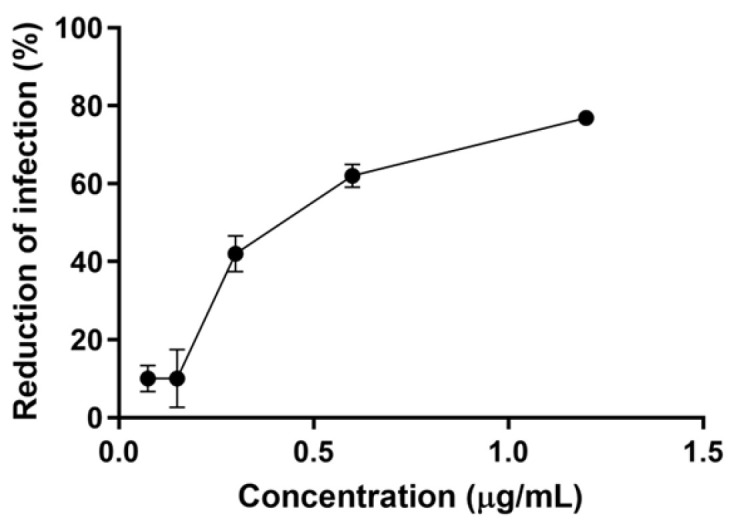
Leishmanicidal activity of heliangin against amastigotes of *L. amazonensis*. Mammalian cells (Raw 264.7) infected with *L. amazonensis* promastigotes were incubated for 48 h with serial dilutions of the compounds. The number of amastigotes was counted using an optical microscope (Carl Zeiss, Jena, Germany) with a 100X oil immersion objective. The percentage reduction in infection (% RI) was calculated as (Survival rate of the treated group/Survival rate of the untreated group) ×100. The median inhibitory concentration (IC_50_) was derived from the dose–response sigmoidal curves using GraphPad Prism version 6 (GraphPad Software Inc., Dotmatics, Boston, MA, USA). The values represent the mean ± SD from three independent experiments conducted in duplicate.

The activity of each compound against the intracellular forms of *L. amazonensis* was subsequently assessed. Heliangin exhibited potent activity against intracellular amastigotes, with an IC_50_ of 0.3 μg/mL (0.8 µM) (Figure 3). In contrast, glaucolide A showed no significant activity against intracellular amastigotes at the highest tested concentration, with an IC_50_ value greater than 3.2 μg/mL (>6.9 μM). The IC_50_ value for amphotericin B was <0.004 μg/mL (Table 1).

### 2.4. Cytotoxicity

The in vitro cytotoxic effects of heliangin and glaucolide A were evaluated on the RAW 264.7 cells. Heliangin exhibited a CC_50_ of 1.5 μg/mL (4.1 µM), while glaucolide A presented a CC_50_ of 3.4 μg/mL (7.3 µM). The reference drug, amphotericin B, showed a CC_50_ of 0.05 µM. CC_50_ values and selectivity indexes are shown in Table 2.

### 2.5. Treatment of L. amazonensis-Infected Mice with Heliangin Alone and in Combination with Glucantime

**Figure 4:**
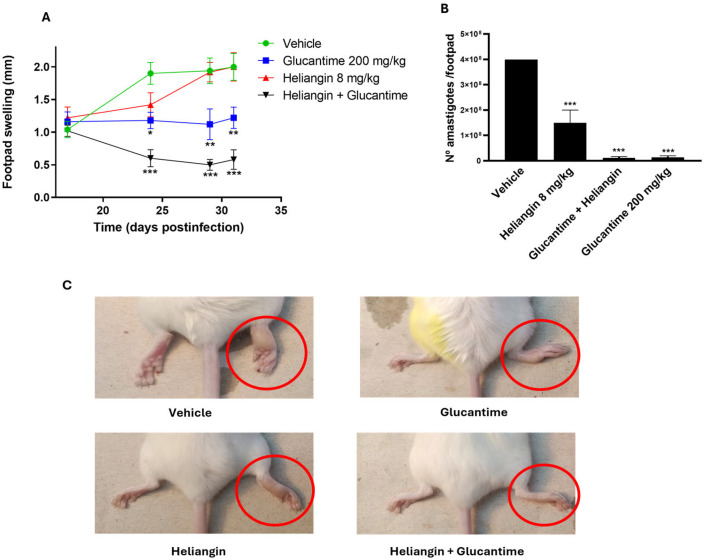
Antileishmanial effect of heliangin alone and in combination with Glucantime in BALB/c mice infected with *L. amazonensis*. (**A**) Footpad swelling (mm) over time in days p.i. (**B**) Parasitic load. (**C**) Macroscopic observations of the lesion size on day 31 p.i. * Statistical differences compared to the vehicle-treated group: *p* < 0.05 (*), *p* < 0.01 (**) and *p* < 0.001 (***).

The antileishmanial effect of heliangin was assessed both alone and in combination with the reference drug Glucantime in BALB/c mice infected with *L. amazonensis*. During the treatment period, the animals treated with the combination of heliangin and Glucantime exhibited lower footpad swelling (*p* < 0.001) compared to those treated with the vehicle. From day 29 post-infection, footpad swelling in the combination-treated group was significantly lower than in the Glucantime-treated group (*p* < 0.05) (Figure 4A). In addition, the parasite burden in animals treated with heliangin was significantly lower (*p* < 0.001) than in the control group; even more, the parasite burden in the combination group was significantly reduced compared to the control group. On the other hand, no differences were observed between the combination group and the mice treated with the reference drug, Glucantime (*p* > 0.05) (Figure 4B). Macroscopic observations on day 31 post-infection are shown in Figure 4C. Importantly, no cases of mortality or weight loss were observed.

## 3. Discussion

In the present study, the crude dichloromethane extracts from two Asteraceae species, *Helianthus tuberosus* and *Vernonathura squamulosa,* were evaluated for their antiprotozoal effects against *L. amazonensis*. In this context, the organic extracts of these species exhibited significant in vitro activity against *L. amazonensis* promastigotes at a concentration of 100 μg/mL, with the organic extract of *H. tuberosus* being the most active against the protozoan.

Two STLs were isolated and identified from *H. tuberosus* and *V. squamulosa* organic extracts. Heliangin and glaucolide A were obtained from *H. tuberosus* and *V*. *squamulosa,* respectively. Previous studies have documented the presence of heliangin in *H. tuberosus* [19] as well as other heliangolides that have shown antiprotozoal activity: 4,15-iso-atriplicolide tiglate, 4,15-iso-atriplicolide methacrylate and isobutyrate, and heliantuberolide-8-O-tiglate [12]. Heliangin has also been reported in other Asteraceae such as *Calea rotundifolia* [17], *Eupatorium kiirunense* [20], *Bejaranoa semistriata*, *Viguiera eriophora*, and *V. puruana* [21]. Similarly, glaucolide A has been reported in *V. squamulosa* aerial parts [22] and has also been identified in the aerial parts of *Bothriocline amplifolia* [23] and other species of the *Vernonia* genus including *V. nudiflora* [24] and *V. polyanthes* [25].

Heliangin and glaucolide A exhibited activity against promastigotes of *L. amazonensis*, with the former being the most active. Furthermore, heliangin was effective against amastigotes, the clinically relevant stage of the parasite, whereas glaucolide A showed no significant activity against this intracellular form at the highest concentration tested.

Sesquiterpene lactones have consistently demonstrated significant antiprotozoal activity. This bioactivity has been largely attributed to the presence of an α,β-unsaturated γ-lactone ring in the structure. Many biological activities exhibited by sesquiterpene lactones can be ascribed to the exomethylene γ-lactone ring, which can react with nucleophiles, particularly sulfhydryl groups in proteins, via a Michael-type addition reaction [26].

Both heliangin and glaucolide A are classified as germacranolides based on their carboxylic skeletons. Heliangin is categorized as a heliangolide-type sesquiterpene lactone, while glaucolide A is classified as a glaucolide-type. When comparing the antiprotozoal activities of heliangin and glaucolide A against the promastigotes and amastigotes of *L. amazonensis*, it can be inferred that the exocyclic double bond in heliangin plays an important role in its anti-*Leishmania* activity. In contrast, glaucolide A, characterized by a γ-lactone ring with an endocyclic double bond, exhibited lower leishmanicidal activity in comparison with heliangin. Sesquiterpene lactones with endocyclic systems such as glaucolides tend to be less active in comparison with those that present an exomethylene in the α,β-unsaturated γ-lactone structure [27]. In this sense, the results obtained for glaucolide A are in accordance with those reported by Sosa et al. [28], who described the activity against promastigotes of *L. amazonensis* and *L. braziliensis* of seventeen sesquiterpene lactones isolated from species of the tribe Vernonieae, including glaucolide A. This compound showed IC_50_ values of 16.2 ± 0.14 μg/mL (35.1 ± 0.30 μM) and 10.8 ± 0.46 μg/mL (23.4 ± 1.00 μM) against promastigote forms of both *L. amazonensis* and *L. braziliensis*, respectively [28]. Glaucolide-type sesquiterpene lactones exhibited moderate leishmanicidal activity against both *L. braziliensis* promastigotes and *L. amazonensis.*

Different STLs have demonstrated activity against *L amazonensis* amastigotes. Parthenolide was active against the intracellular form of the parasite, with an IC_50_ of 0.81 μg/mL [29]. Artemisinin displayed an IC_50_ value of 15.0 μM on amastigotes with low cytotoxicity in macrophages (200 μM), exhibiting a high selectivity index (>13) [30]. Other STLs, 8-epi-xanthatin-1β,5β-epoxide and inuloxin A isolated from *Inula viscosa*, demonstrated IC_50_ values of 6.98 and 0.64 μM, respectively, against the amastigote stage of *L. amazonensis* [31]. In relation to heliangolides, 11,13-dihydroxy-calaxin, a furanoheliangolide isolated from *Calea pinnatifida*, exhibited promising leishmanicidal activity with an IC_50_ of 8.30 µM against amastigotes of *L. amazonensis* [32].

The difference in activity and selectivity between heliangin and glaucolide A suggests that heliangin may be a more promising candidate for further investigations. In this sense, heliangin was selected for its evaluation on a mice model of experimental cutaneous leishmaniasis [33]. The antileishmanial effect of heliangin alone and in combination with Glucantime was evaluated in BALB/c mice infected with *L. amazonensis*. A significant decrease in lesion size was observed in the group treated with the combination of heliangin and Glucantime compared to the group treated with vehicle; however, the group treated with heliangin alone showed no significant differences in lesion size compared to the control group. Nevertheless, the group treated with heliangin showed a significant reduction in parasite load compared to the control group; although, its efficacy did not exceed that of glucantime.

Heliangin has shown anti-inflammatory activity in reducing the levels of IL-6 and TNF-α [34]. Previous studies demonstrated that human anti-TNF-α antibodies alone do not reduce the lesions size caused by *Leishmania mayor*; however, the combination of antibody and paromomycin had smaller lesions than animals treated with paromomycin alone at the end of the experiment [35]. Heliangin has shown anti-inflammatory properties by inhibiting the expression of adhesion molecules, including ICAM-1, VCAM-1, E-selectin, and MCP-1, as well as the phosphorylation of NF-κB and IκBα in TNF-α-stimulated endothelial cells. These mechanisms would indicate that heliangin may play a role in modulating inflammatory responses [21]. In addition to its inherent antiprotozoal activity, heliangin may also regulate inflammatory processes, providing a beneficial effect against parasite infections.

Determining the mechanism of action and identifying the potential molecular targets of antiprotozoal drug candidates are critical aspects of drug discovery. Sesquiterpenes exhibit promising anti-Leishmania activity through various possible mechanisms. It has been demonstrated that this type of terpenoid induces cell death by apoptosis, necrosis, autophagy, and the disruption of the cell membrane. These processes result in alterations of the cellular metabolism and structure, thereby compromising the survival and proliferation of the parasite [36]. For instance, the STL artemisinin has been shown to decrease mitochondrial membrane potential, reduce ATP levels, and induce oxidative stress, leading to mitochondrial dysfunction and parasite death [36]. Similarly, dihydroartemisinin showed greater efficacy than artemisinin in targeting *L. braziliensis*, inducing oxidative stress, mitochondrial dysfunction, and the inhibition of crucial parasite proteins, the loss of membrane integrity, heme alkylation, and the covalent interaction with biological molecules [37]. The STL xanthatin, isolated from *Xanthium strumarium*, has demonstrated anti-*Leishmania* activity. This compound interferes with sugar and carbon metabolism, thereby reducing parasite virulence [38]. Additionally, transmission electron microscopy analysis revealed that the STLs calein and calaleactone C damage mitochondrial and nuclear structures in the parasites, contributing to cell death [39].

The sesquiterpene lactones 8-epi-xanthatin-1β,5β-epoxide and inuloxin A, extracted from *Inula viscosa*, exhibited distinct effects on the *L. amazonensis* parasite. Exposure of *L. amazonensis* to 8-epi-xanthatin-1β,5β-epoxide resulted in a pronounced condensation of the chromatin structure, together with characteristics indicative of cell death, such as the penetration of propidium iodide through the cell membrane. On the other hand, inuloxin A caused significant mitochondrial depolarization and a substantial decrease in ATP levels and altered the integrity and permeability of the plasma membrane in *L. amazonensis.* In contrast, 8-epi-xanthatin-1b,5b-epoxide increased the generation of reactive oxygen species and affected the plasma membrane permeability in this parasite [31]. Additionally, the bioactive STLs mexicanin I, dehydroleucodine and psilostachyin promoted an increase in reactive oxygen species (ROS) production within the parasites [40].

It is presumed that sesquiterpene lactones may exert their leishmanicidal effects through the generation of an oxidative environment within the parasite. These compounds are known to undergo Michael-type addition with sulfhydryl groups, potentially inhibiting the activity of crucial enzymes involved in the parasite’s defense against oxidative stress. This mechanism could result in an increase in reactive oxygen species and parasite damage stemming from a disruption in the redox balance [40]. Given these observations, it is plausible that heliangin may share similar mechanisms, including mitochondrial dysfunction and oxidative stress. In this context, further investigations are needed to identify the molecular target(s) of heliangin.

In this study, we have evaluated the in vitro activity of heliangin on *L. amazonensis* and the assessment of this compound on an animal model of cutaneous leishmaniasis. Further studies should be carried out in order to complete preclinical study. For instance, the identification and validation of molecular targets is a critical step in the drug development process. In addition, the evaluation of other doses and routes of administration as well as long-term experiments will also be considered. These studies aim to assess the sustained efficacy of heliangin-based treatments and provide valuable insights into the long-term durability of the drug’s effects. Over the last decade, notable advancements have been made in the treatment and preclinical development of drugs for visceral leishmaniasis. However, research and development of new drugs and treatments for cutaneous leishmaniasis have lagged [33].

Moreover, after the identification of a hit (promising compound), lead selection and the optimizing of the lead should be performed. The drug’s absorption, distribution, metabolism, and excretion; its potential benefits and mechanisms of action; potential side effects or toxicity; interactions with other drugs and treatments; and its comparative effectiveness to similar drugs have been addressed by the FDA’s drug development guidelines and are considered crucial before advancing a drug to the clinical stage [41].

In order to enhance the selectivity index (SI) of heliangin, we aim to investigate strategies to reduce its toxicity by combining it with a reference drug. Previous studies have highlighted the potential of combining compounds to modulate their effects and optimize their therapeutic profiles [42,43]. Similarly, natural products have long been recognized for their structural diversity and therapeutic potential, and modifying these natural compounds has proven to be an effective strategy in drug development. By exploring heliangin-derived compounds and their derivatives, we can create new entities with improved pharmacological properties, which aligns with our approach of improving heliangin’s therapeutic profile [44].

Cutaneous leishmaniasis is caused by different Leishmania species, which are mainly present in the Americas, producing skin lesions that can result in permanent scarring and serious disability or stigma. This study represents a preliminary investigation aimed at assessing its antiparasitic effect against *L. amazonensis*, one of the species, together with *L. braziliensis*, which is responsible for cutaneous leishmaniasis in Argentina. In this regard, in the future, the evaluation of the effect of heliangin against other *Leishmania* species that are endemic to our region will be considered.

## 4. Materials and Methods

### 4.1. Plant Material

The aerial parts (leaves and flowers) of *Helianthus tuberosus* L. (Asteraceae) and *Vernonanthura squamulosa* (Hook. & Arn.) H. Rob. (Asteraceae) were collected in March 2019 in Escobar, Buenos Aires Province, Argentina, and in September 2021 in San Javier, Tucumán Province, Argentina, respectively. Botanical identification of the plant material was performed and voucher specimens (BAF 14870 for *H. tuberosus* and BAF 16125 for *V. squamulosa*) were deposited at the Museo de Farmacobotánica, Facultad de Farmacia y Bioquímica, Universidad de Buenos Aires. The plant material was air-dried at room temperature and preserved in labeled containers that included the species name, herbarium number, collection date, collection site, and the specific plant part collected. The labeled containers were stored in a cool, dry, and light-protected environment.

### 4.2. Parasites

Promastigotes of *Leishmania amazonensis* (MHOM/BR73/M2269 reference strain) were isolated from lesions in mice and maintained in Difco blood agar (USMARU) medium supplemented with 20% defibrinated rabbit blood plus sterile proline-balanced salts solution (PBSS) with 100 U/mL of penicillin and 50 μg/mL of streptomycin (P-S) at 24 °C. RPMI-1640 medium (Gibco, Grand Island, NY, USA) supplemented with 10% (*v*/*v*) heat-inactivated fetal bovine serum (FBS) and P-S was utilized for the in vitro assays.

### 4.3. Preparation of Crude Dichloromethane Extracts

The air-dried aerial parts of *H. tuberosus* and *V. squamulosa* (200 g) were extracted by maceration with dichloromethane (2 L) (Sintorgan, Buenos Aires, Argentina) at room temperature for 5 min, repeating the process twice. The organic extracts were then collected, filtered, and the solvent was removed under reduced pressure, resulting in yields of 1.5% and 2.9%, respectively. Crude dichloromethane extracts of *H. tuberosus* and *V. squamulosa* were evaluated in vitro to determine their activity against *L. amazonensis* promastigotes.

### 4.4. Fractionation and Purification of the Organic Extracts

#### 4.4.1. Fractionation of the Crude Dichloromethane Extract of *Helianthus tuberosus*

The *H. tuberosus* crude dichloromethane extract (3.6 g) was fractionated by open-column chromatography using silica gel 60 (0.063–0.2 mm, Macherey-Nagel, Dueren, Germany) and eluted with a gradient of hexane (Hx) and ethyl acetate (EtOAc) (Sintorgan, Buenos Aires, Argentina). Fifty fractions of 200 mL each were collected (2 × Hx 100%; 6 × Hx: EtOAc 9:1; 13 × Hx: EtOAc 7:3; 13 × Hx: EtOAc 5:5; 9 × Hx: EtOAc 3:7; 7×EtOAc 100%). The fractions were analyzed by Thin Layer Chromatography (TLC) [stationary phase: Silicagel F254 (Merck, Germany); mobile phase: Hx:EtOAc (1:1)] using UV light (254 and 366 nm) and anisaldehyde-sulfuric acid (Sigma-Aldrich, St. Louis, CA, USA) reagent for visualization. From fractions 27 to 32, a white solid was obtained (compound A) (12 mg).

#### 4.4.2. Obtention of *Vernonanthura squamulosa* Dewaxed Dichloromethane Extract

The crude dichloromethane extract of *V. squamulosa* (5.8 g) was suspended in 87 mL of a 70:30 ethanol–water mixture and then successively partitioned with hexane (3 × 50 mL) and dichloromethane (CH_2_Cl_2_) (3 × 50 mL). The hexane phase was set aside without further processing. The dewaxed dichloromethane phase was dried over anhydrous sodium sulfate (Biopack, Ciudad Autónoma de Buenos Aires, Argentina), filtered, and concentrated to dryness using a rotary evaporator (R-134, Buchi, Flawil, Switzerland). During the concentration process, an impure solid was obtained (803 mg). The solid was washed with ether (3 × 50 mL) (Sintorgan, Buenos Aires, Argentina) to afford a crystalline compound (compound B, 710 mg).

### 4.5. Purity of Isolated Compounds

The purity of the isolated compounds was assessed using HPLC/DAD with a Waters instrument (Milford, CT, USA) equipped with a photodiode array detector (Waters 2996), a Rheodyne injection valve (20 μL), a Waters 600 controller, a Delta 600 pump (Milford, MA, USA), and an in-line degasser. A reversed-phase Agilent Eclipse Plus C-18 column (250 mm × 4.6 mm, 5 μm) (Agilent, Santa Clara, CA, USA) was utilized, with the photodiode array detector set at 210 nm. The isolated compounds were dissolved in a 1:1 mixture of acetonitrile and water at concentrations of 0.5 mg/mL. Prior to elution, the solutions were filtered through a nylon filter (0.45 μm, Agilent). A gradient elution was carried out using water (A) and acetonitrile (B), progressing from 35% B to 95% B over 30 min. The flow rate was maintained at 1.0 mL/min. Ultrapure water (Milliq) and J.T. Baker acetonitrile (HPLC grade) (Phillipsburg, KS, USA) were used to prepare the mobile phase. Chromatograms were recorded and analyzed using Empower Pro 3 software (https://www.waters.com/waters/en_US/Empower-3-Chromatography-Data-Software/nav.htm?cid=513188&locale=en_US accessed on 10 November 2024), allowing for the determination of compounds’ purity through the integration of the detected peaks.

### 4.6. Identification of the Compounds

The identities of the isolated compounds, A and B, were determined using proton nuclear magnetic resonance (^1^H-NMR), carbon nuclear magnetic resonance (^13^C-NMR) as well as heteronuclear single quantum correlation (HSQC), heteronuclear multiple bond correlation (HMBC), and correlated spectroscopy (COSY) on a Bruker Avance 600 (Billerica, MA, USA) (600 MHz in CDCl_3_). Additionally, electron impact mass spectrometry (EI-MS) was employed. Structural elucidation was confirmed by comparing the experimental spectra with data in the literature.

### 4.7. In Vitro Assay of Leishmanicidal Activity

#### 4.7.1. In Vitro Activity Against Promastigotes of *L. amazonensis*

The in vitro activity of the crude dichloromethane extract from *H. tuberosus* and *V. squamulosa*, as well as the isolated compounds A and B, was evaluated against promastigotes of *L. amazonensis*. Parasites (5 × 10⁵ per well) were added into 96-well plates and treated with the crude dichloromethane extract (10 µg/mL and 100 µg/mL) and serial dilutions (2–50 µg/mL) of the compounds in RPMI medium. The plates were incubated at 24 °C for 48 h. After incubation, 50 µL of XTT reagent (0.3 mg/mL) was added to each well, followed by an additional 4 h of incubation. Parasite viability was assessed by measuring absorbance at 450 nm using a microplate reader (Tecan, Mannedorf, Switzerland). A dose–response sigmoidal curve was constructed for compounds A and B to determine the median inhibitory concentrations (IC_50_) using GraphPad Prism 6. Amphotericin B was used as positive control.

#### 4.7.2. In Vitro Activity Against Amastigotes of *L. amazonensis*

The in vitro activity of the isolated compounds was evaluated against the intracellular forms of *L. amazonensis*. Mammalian cells (Raw 264.7) were infected with *L. amazonensis* promastigotes in the stationary phase at a ratio of 15 parasites per cell. After overnight infection at 34 °C and 5% CO_2_, non-infecting parasites were washed out, and the culture was incubated until amastigotes were observed within the parasitophorous vacuole. Infected cells were then seeded (1.15 × 10^4^ per well) in a 16-well plate (LAB Tek, Nunc, USA) and incubated for 4 h under the same conditions. After incubation time, serial dilutions (1 µg/mL–20 µg/mL) of the compounds were added in duplicate and incubated for an additional 48 h. The culture medium was subsequently removed, and the cells were fixed and stained using Stain 15 (BIOPUR, Rosario, Argentina). The number of amastigotes was counted using an optical microscope with a 100× oil immersion objective. The percentage reduction in infection (% RI) was calculated using the following formula:

%RI = (Survival rate of the treated group/Survival rate of the untreated group) ×100

where

Survival rate = % Infected macrophages × (amastigotes number/macrophages)

The mean inhibitory concentration (IC_50_) was calculated from the dose–response sigmoidal curves using GraphPad Prism version 6. Amphotericin B (Aldrich, St. Louis, MO, USA) was used as positive control.

### 4.8. In Vitro Assay for Cytotoxicity

The cytotoxic effects of the isolated pure compounds A and B were evaluated in vitro using RAW 264.7 mammalian cells. The assessment of cytotoxicity was conducted using the XTT method. RAW 264.7 cells (4 × 104 cells per well) were seeded in sterile 96-well plates and cultured at 37 °C in a 5% CO_2_ atmosphere. After 4 h, the culture medium was removed, and serial dilutions of the pure compounds and the reference drug (Amphotericin B, Aldrich, St. Louis, MO, USA), ranging from 0.1 to 100 μg/mL, were added. DMSO was included as a viability control and the cells were incubated for an additional 48 h. Following this incubation, 50 µL of XTT reagent (0.3 mg/mL) was added to each well, and the plate was incubated for an additional 4 h under the same conditions. Cell viability was measured using a microplate reader (Tecan, Mannedorf, Switzerland) at a wavelength of 450 nm. The CC_50_, defined as the concentration that induces cell death in 50% of the cells, was calculated using a dose–response sigmoidal curve constructed with GraphPad Prism 6 (GraphPad Software Inc., Dotmatics, Boston, MA, USA). Amphotericin B was included in the assay.

### 4.9. Animal-Based Model of L. amazonensis Infection

The in vivo study was performed using healthy female BALB/c mice raised in the animal facility of the Instituto de Patología Experimental (UNSA-CONICET), Universidad Nacional de Salta, Argentina. Groups of five 6-week-old mice were infected by an intradermal route with 1 × 10^6^ *L. amazonesis* promastigotes in the stationary phase in the right footpad (FP). Three weeks post-infection, the mice were randomly assigned to one of four treatment groups. Treatments were administered every 48 h for 12 days: one group received heliangin (8 mg/kg) intralesionally, another received Glucantime (Aventis) (200 mg/kg) intraperitoneally, a third group received a combination of both treatments (heliangin and Glucantime) at the same doses and routes, and the control group was treated with the vehicle solution (DMSO in PBS, pH 7.2). Treatment efficacy was assessed by measuring the footpad swelling in the infected FP of the animals once a week using a digital caliper and quantifying the parasite loads by the LD assay [45]. Briefly, the infected right footpad was excised and weighed; then the tissue was homogenized with 5 mL of complete RPMI using a glass grinder. After serial dilutions in 96-well plates, the samples were incubated at 23 °C for 14 days, and the number of viable promastigotes was determined from the highest dilution at which the parasite could grow.

### 4.10. Ethical Statement

Animal experiments received approval from the Ethics Review Board of the Institutional Commission for the Care and Use of Laboratory Animals (CICUAL) of Facultad de Ciencias de la Salud, Universidad Nacional de Salta (Argentina), under No. 12.161/2023, and were carried out in accordance with the Guide for the Care and Use of Laboratory Animals published by the National Research Council [46].

### 4.11. Statistical Analysis

Statistical analysis was performed using GraphPad Prism 6.0 software. In the in vitro assays, the IC_50_ and CC_50_ values were calculated using nonlinear regression. In the in vivo assay, an analysis of variance (ANOVA) followed by a post hoc Bonferroni test was used for comparisons between groups. Values of *p* < 0.05 were considered statistically significant. Additionally, different levels of significance were identified: *p* < 0.01 and *p* < 0.001.

## 5. Conclusions

In this work, the isolation and antileishmanial activity of the sesquiterpene lactones heliangin and glaucolide A isolated from *Helianthus tuberosus* and *Vernonanthura squamulosa*, respectively, was described. Heliangin demonstrated the highest in vitro activity against both promastigotes and amastigotes of *L. amazonensis*. In this context, an in vivo study of this sesquiterpene lactone was conducted using an animal model of cutaneous leishmaniasis, showing promising results when combined with the reference drug Glucantime. Further studies will be conducted in the future in order to elucidate the mode of action of this compound and its effect on other *Leishmania* species.

## Figures and Tables

**Table 1:** Antileishmanial activity on promastigotes and amastigotes of *L. amazonensis* of the isolated compounds.

Compounds	IC_50_ ± SD (μg/mL) (μM)	
	Promastigote	Amastigote
Heliangin	3.4 ± 0.1 (9.3 ± 0.4)	0.3 ± 0.1 (0.8 ± 0.3)
Glaucolide A	21.7 ± 1.5 (46.7 ± 3.1)	>3.2 (>6.9)
Amphotericin B	0.1 ± 0.09 (0.1 ± 0.09)	<0.004 (<0.004)

**Table 2:** Citotoxicity on RAW 264.7 mammalian cells and selectivity indexes of the isolated compounds.

Compounds	CC_50_ ± SD (μg/mL) (µM)	Selectivity Index	
	Macrophages	Promastigotes	Amastigotes
Heliangin	1.5 ± 0.2 (4.1 ± 0.5)	0.4	5.1
Glaucolide A	3.4 ± 0.3 (7.3 ± 0.6)	0.2	<1
Amphotericin B	0.05 ± 0.03 (0.05 ± 0.03)	0.5	>12.5

## Data Availability

The original contributions presented in the study are included in the article, and further inquiries can be directed to the corresponding authors.

## Supplementary Materials

The following supporting information can be downloaded at: https://www.mdpi.com/article/10.3390/molecules30051039/s1: Figure S1: HPLC chromatogram of A (heliengin); Figure S2: HPLC chromatogram of B (glaucolide A); Figure S3: ^1^H-NMR of compound A (heliengin); Figure S4: ^13^C-NMR of compound A (heliengin); Figure S5: HSQC of compound A (heliengin); Figure S6: HMBC of compound A (heliengin); Figure S7: COSY of compound A (heliangin); Figure S8: ^1^H-NMR of compound B (glaucolide A); Figure S9: ^13^C-NMR of compound B (glaucolide A); Figure S10: HSQC of compound B (glaucolide A); Figure S11: HMBC of compound B (glaucolide A); Table S1: NMR data of compound A (heliangin) in CDCl_3_ at 600 MHz; Table S2: NMR data of compound B (glaucolide A) in CDCl_3_ at 600 MHz.
