# Supplementary material for: Bioactive Compounds with Leishmanicidal Potential from Helianthus tuberosus and Vernonanthura squamulosa
Source: Molecules. 2025 Feb 24;30(5):1039. doi: 10.3390/molecules30051039 (PMC11901713; doi:10.3390/molecules30051039)
Supplement: Supplementary file 1 [file molecules-30-01039-s001.zip › molecules-3428945-supplementary.pdf]

Bioactive Compounds with Leishmanicidal Potential from *Helianthus tuberosus* and *Vernonanthura squamulosa*

Rachel Nápoles Rodríguez [1], María Laura Arreguez [2], Aldana M. Corlatti [1,3], Hernán G. Bach [4], César A. N. Catalán [5], Laura C. Laurella [1,3], Paola A. Barroso [2,*] and Valeria P. Sülsen [1,3,*]

[1] CONICET-Universidad de Buenos Aires, Instituto de Química y Metabolismo del Fármaco (IQUIMEFA), Autonomous City of Buenos Aires C1113AAD, Argentina; rnapolesrodriguez@gmail.com (R.N.R.); aldanamalencorlatti@gmail.com (A.M.C.); c.laurella@docente.ffyb.uba.ar (L.C.L.)
[2] CONICET-Universidad Nacional de Salta, Instituto de Patología Experimental (IPE), Salta A4400, Argentina; arreguezmarialaura@yahoo.com.ar
[3] Universidad de Buenos Aires, Facultad de Farmacia y Bioquímica, Cátedra de Farmacognosia, Autonomous City of Buenos Aires C1113AAD, Argentina; aldanamalencorlatti@gmail.com
[4] Instituto Nacional de Tecnología Agropecuaria, Buenos Aires B1686, Argentina; bach.hernan@inta.gob.ar
[5] Instituto de Química Orgánica, Facultad de Bioquímica, Química y Farmacia, Universidad Nacional de Tucumán, San Miguel de Tucumán T4000INI, Tucumán, Argentina; cancatalan@gmail.com
* Correspondence: barrosopaola75@gmail.com (P.A.B); vsulsen@ffyb.uba.ar (V.P.S.); Tel.: +54 (011) 5287-4272 (V.P.S.)

Supplementary Materials

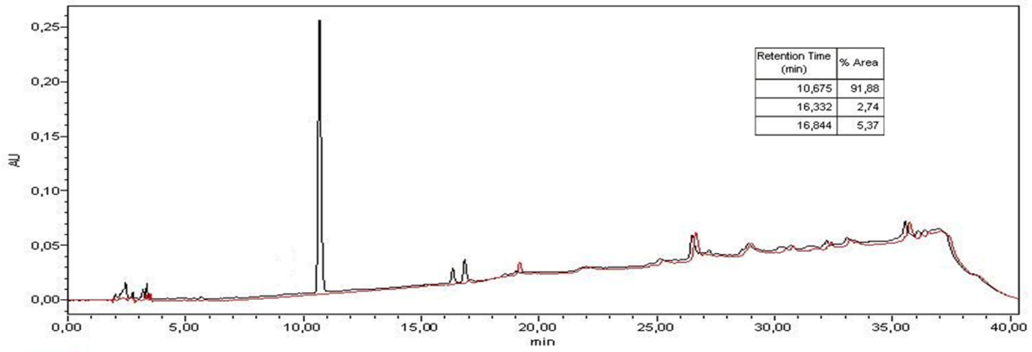

Figure S1. HPLC chromatogram of compound A (Heliangin).

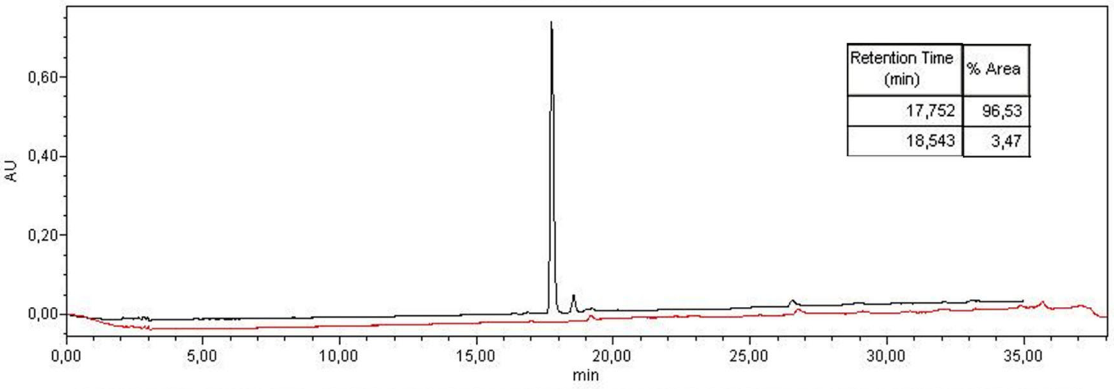

Figure S2. HPLC chromatogram of compound B (Glaucolide A).

Table S1. NMR data of compound A (Heliangin) in $CDCl_3$ at 600 MHz.

cCarbon	δδ $_C$ (ppm)	HH	δ ^{1}H (*J* Hz)	H-H COSY	HMBC
1	60.6	1	2.81dd (10, 4.5)	2a, 2b, 3 (weak)	3, 9a, 2a, 2b, 14, 9b
2	32.5	2a	2.47 ddd (14.9, 4.5, 4.3)	1, 2b, 3	3, 1
		2b	1.75 ddd (14.9, 10, 2.4)	1, 2a, 3	
3	72.4	3	4.51 dd (4.3, 2.4)	2a, 2b	5, 2a, 15, 2b
4	141.6		-	-	6, 3, 15, 2b
5	126.6	5	5.34 (11, 1.2)	6, 15	15, 6, 3, 7
6	74.1	6	6.67 dd (11, 2.2)	5, 7	8, 7
7	48.6	7	2.88 m	6, 13a, 13b, 8 (weak)	6, 13a, 13b, 5, 8, 9a, 9b
8	76.2	8	5.18 ddd (4.5, 2.3, 1.2)	7 (weak), 9a, 9b	6, 9a, 13a, 13b
9	43.1	9a	2.83 dd (14.8, 4.5)	9b, 8	8, 1, 14
		9b	1.33 dd (14.8, 2.3)	9a, 8	
10	58.6	10	-		8, 9a, 9b, 2a, 14, 2b
11	137.3	11	-		6, 13a, 13b, 8
12	169.5	12	-		6, 13a, 13b, 7
13	124.8	13a	6.36 d (2.1)	7	7
		13b	5.76 d (1.9)	7	
14	19.7	14	1.47 s	-	1, 9a, 9b
15	23.0	15	1.82 d (1.2)	5	5
1’	166.7	1’	-	-	3’, 8, 5’
2’	127.8	2’	-	-	5’, 4’
3’	139.0	3’	6.86 qq (7, 1.2)	4’, 5’	4’, 5’
4’	14.6	4´	1.79 d (br) (7)	3´	3’
5’	12.0	5’	1.80 quint (0.9)	3’, 4’	3’

These NMR data agree and complement those reported for heliangin: Saiki P, Yoshihara M, Kawano Y, Miyazaki H, Miyazaki K. Anti-Inflammatory Effects of Heliangin from Jerusalem Artichoke (*Helianthus tuberosus*) Leaves Might Prevent Atherosclerosis. Biomolecules. 2022 Jan 6;12(1):91. doi: 10.3390/biom12010091.

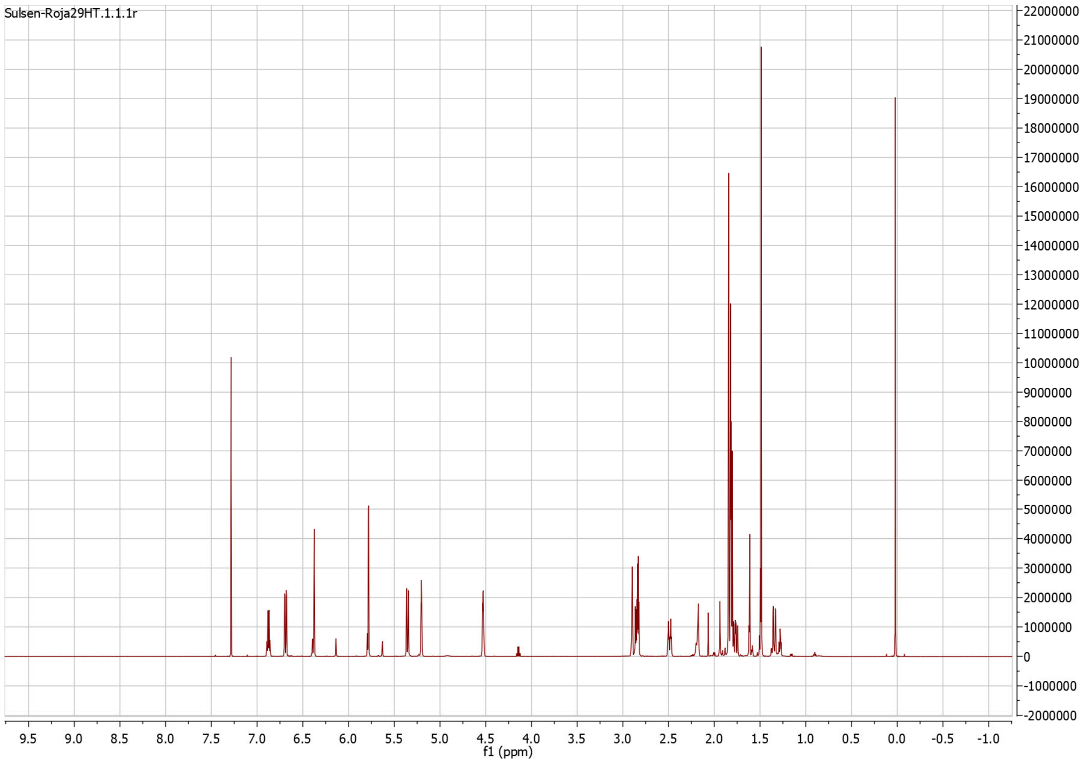

Figure S3. ^{1}H-NMR of compound A (Heliangin).

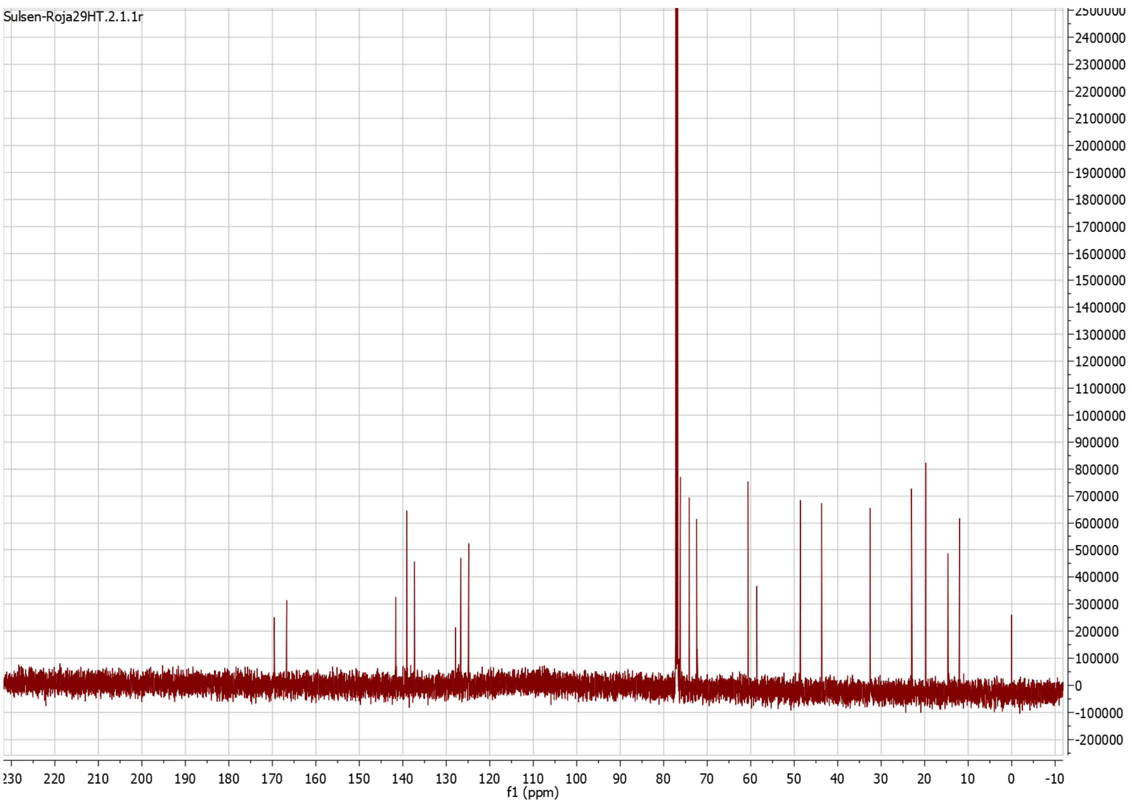

Figure S4. ^{13}C-NMR of compound A (Heliangin).

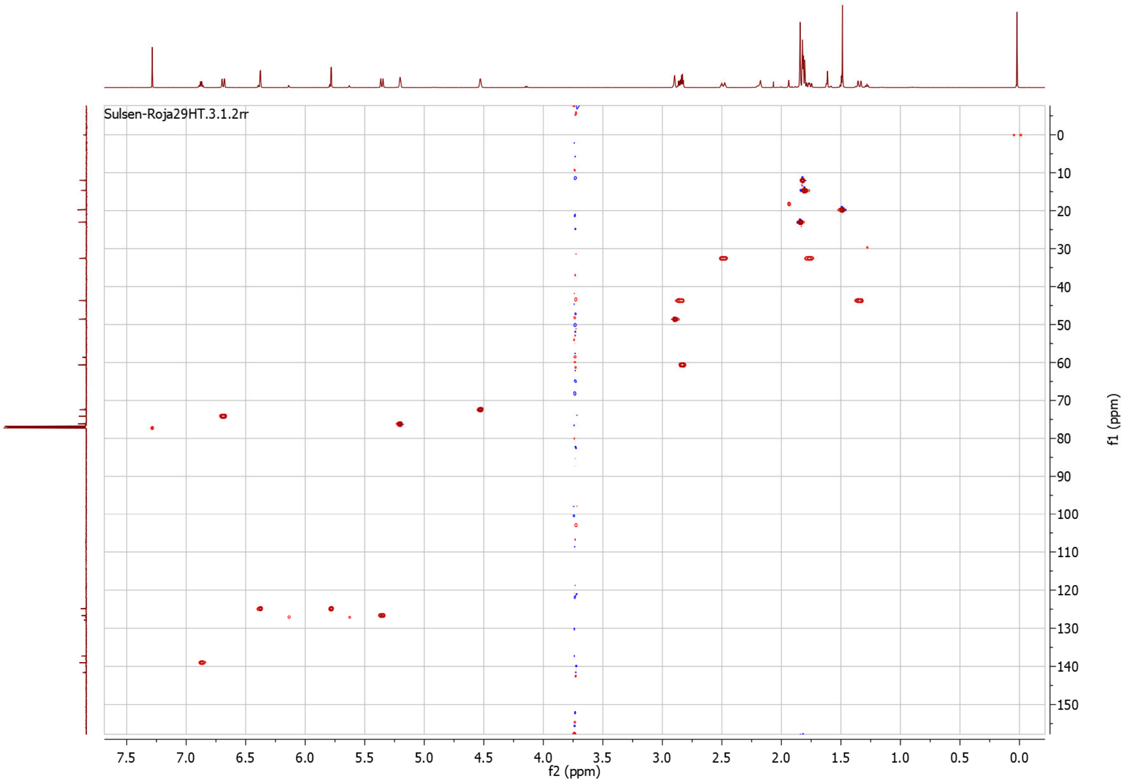

Figure S5. HSQC of compound A (Heliangin).

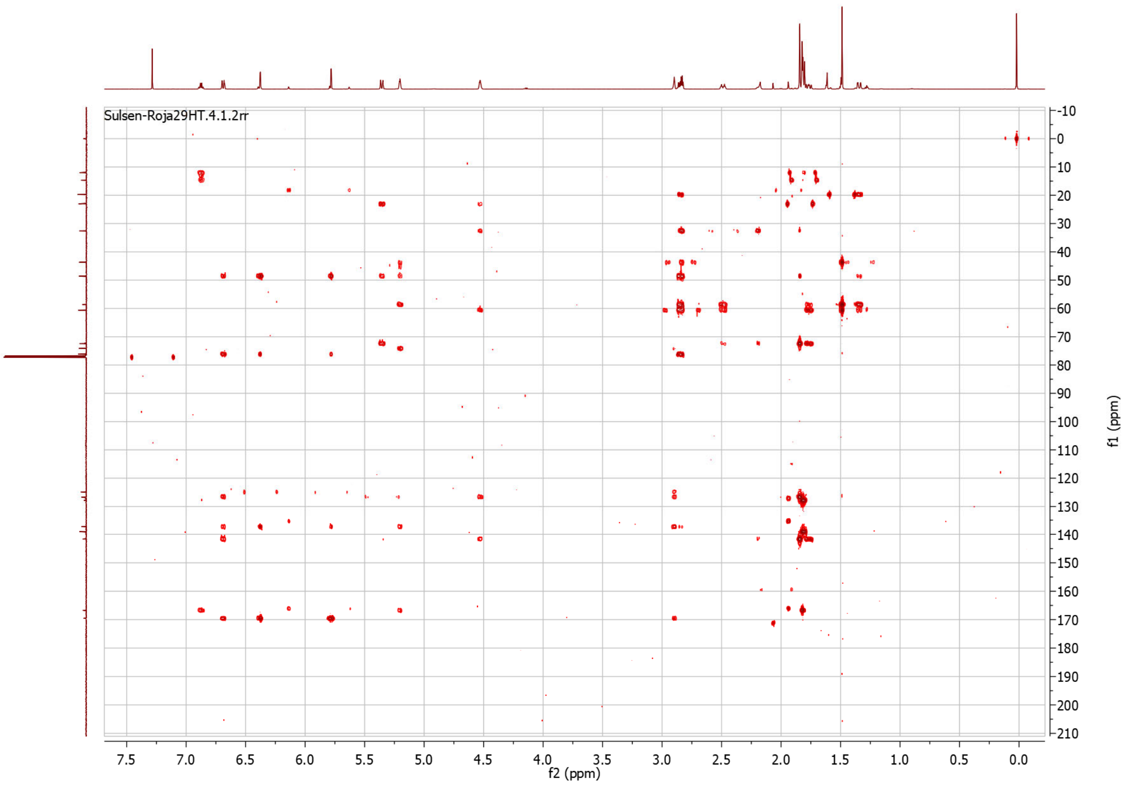

Figure S6. HMBC of compound A (Heliangin).

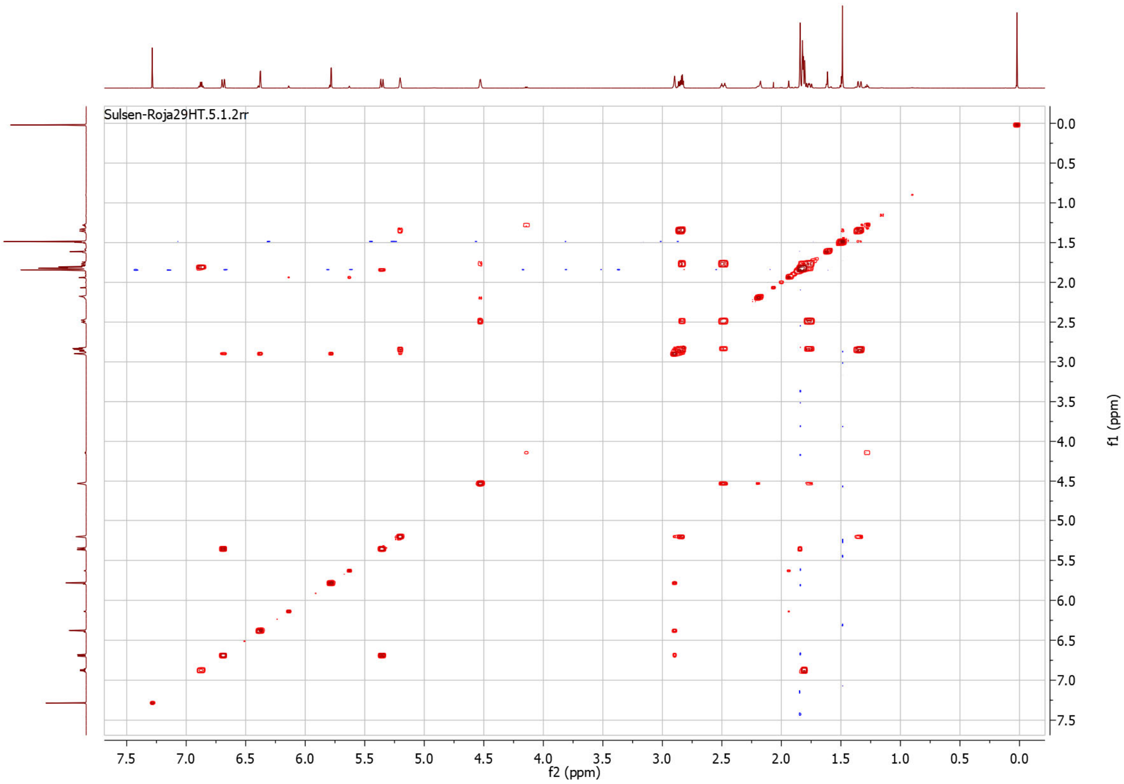

Figure S7. COSY of compound A (Heliangin).

Table S2. NMR data of glaucolide A in $CDCl_3$ at 600 MHz. .

cCarbon	δδ C (ppm)	HH	δ ¹H (*J* Hz)	HMBC
1	206.6 (br)	1	-	-
2	32.8 (br)	2a<br>2b	2.94 (br)<br>2.29 (br)	-
3	31.8 (br)	3a<br>3b	2.61 (br)<br>1.66 (*)	15
4	61.4 (br)	4	-	3b, 6, 15
5	58.9 (br)	5	2.80 (br)	6, 15
6	80.8 (br)	6	4.91 d (9.6)	-
7	163.3	7	-	6, 8
8	64.5 (br)	8	4.85 m (#)	-
9	40.4 (br)	9a<br>9b	2.82 (br)<br>2.29 (br)	-
10	84.8 (br)		-	-
11	125.1		-	13a, 6, 13b
12	169.7		-	-
13	55.0	13a<br>13b	4.84 d (br) (12.1) (#)<br>4.81 d (br) (12.1)	Ac1, Ac2 (methyl)
14	18.9 (br)	14	1.56 (br)	-
15	22.4 (br)	15	1.67 s	-
1’	166.3	1’	-	3’a, 3’b, 4’
2’	134.8	2’	-	3’a, 4’
3’	127.7	3’a<br>3’b	6.16 s<br>5.71 s	4’
4’	18.0	4′	1.94 s (br)	3′a, 3’b
Ac1	170.2, 20.7	Ac1	2.07 s	Ac2 (methyl)
Ac2	170.9, 21.0	Ac2	2.08 s	Ac1 (methyl)

Spectra obtain at room temperature. (*): obscured by intense methyl signal. (#): superimposed signals.

It should be noted that glaucolide A and similar glaucolides in deuterochloroform at room temperature show broad (br) signals due to conformational equilibrium. Our NMR data obtained at room temperature agree with those reported at 57º C if the temperature difference is taken into account. See A. Bardon, C.A.N. Catalan, A.B. Gutierrez, W. Herz (1990). Glaucolides and related sesquiterpene lactones from *Vernonia incana*. *Phytochemistry* 29, 313-315.

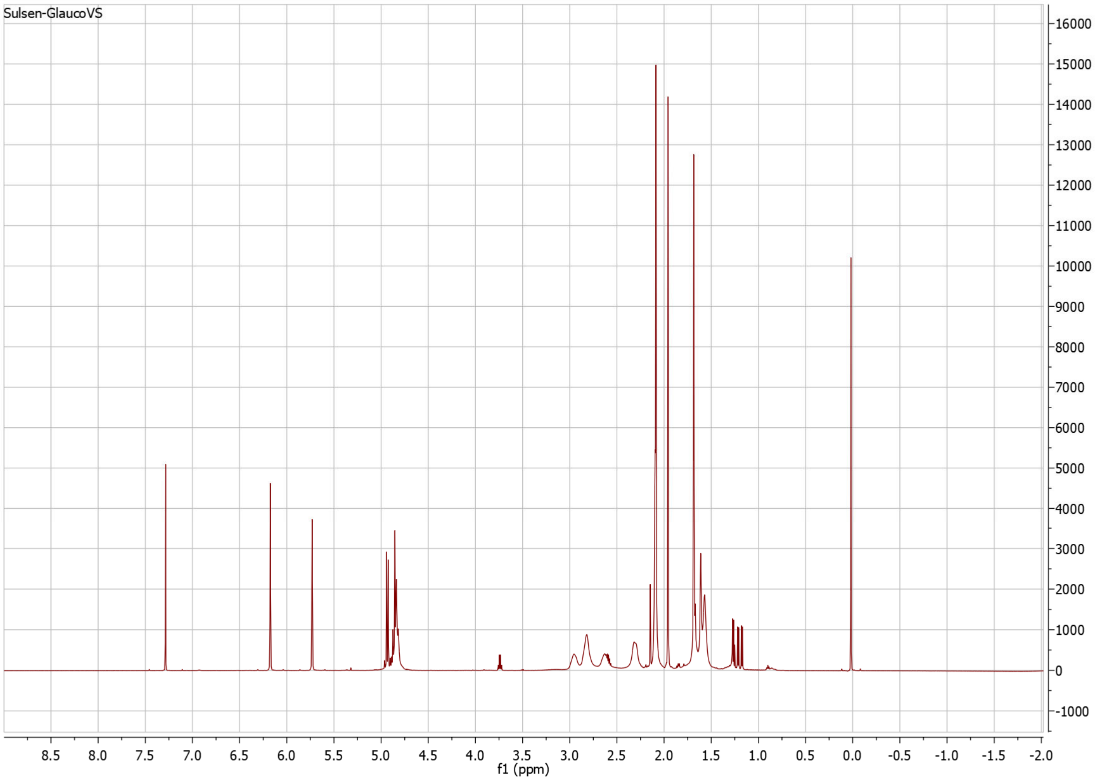

Figure S8. ¹H-NMR of compound B (Glaucolide A).

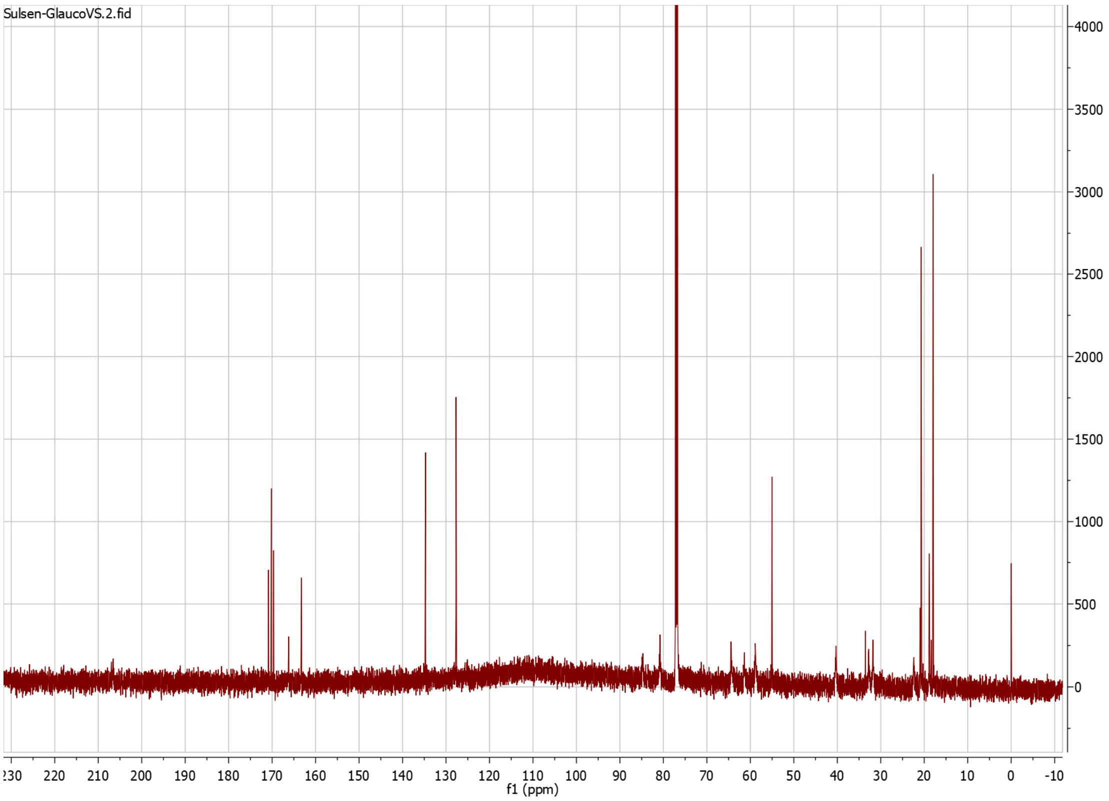

Figure S9. ^{13}C-NMR of compound B (Glaucolide A).

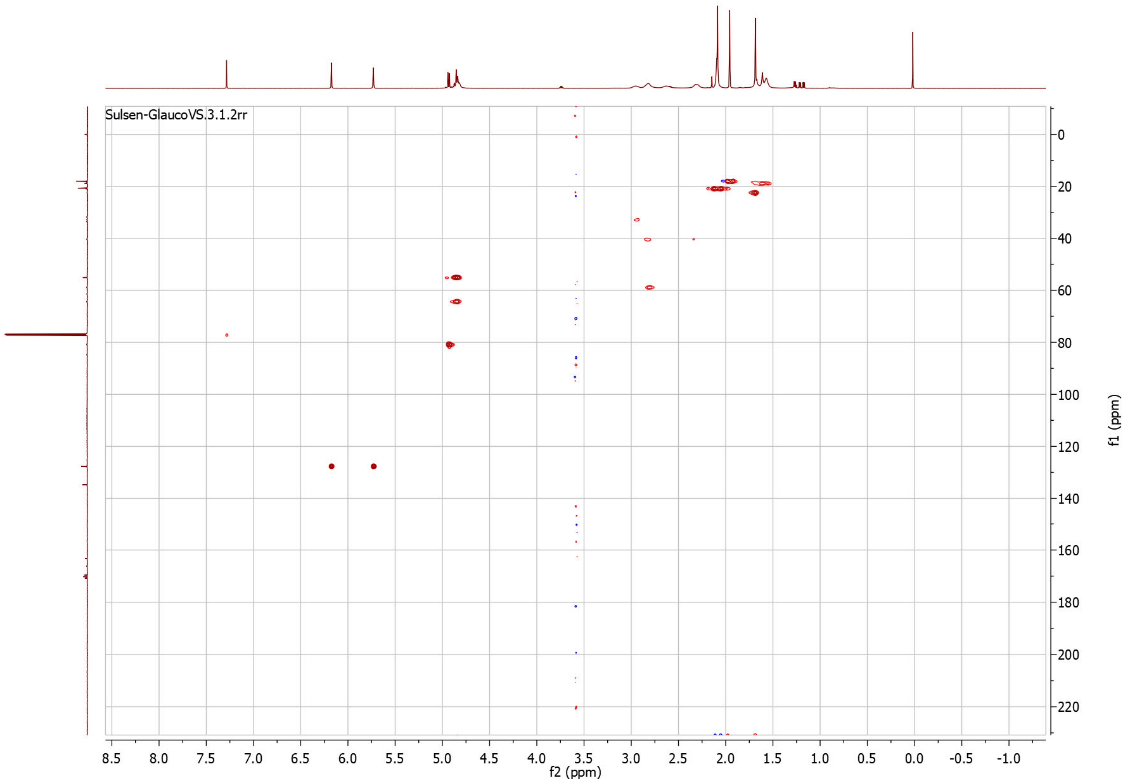

Figure S10. HSQC of compound B (Glaucolide A).

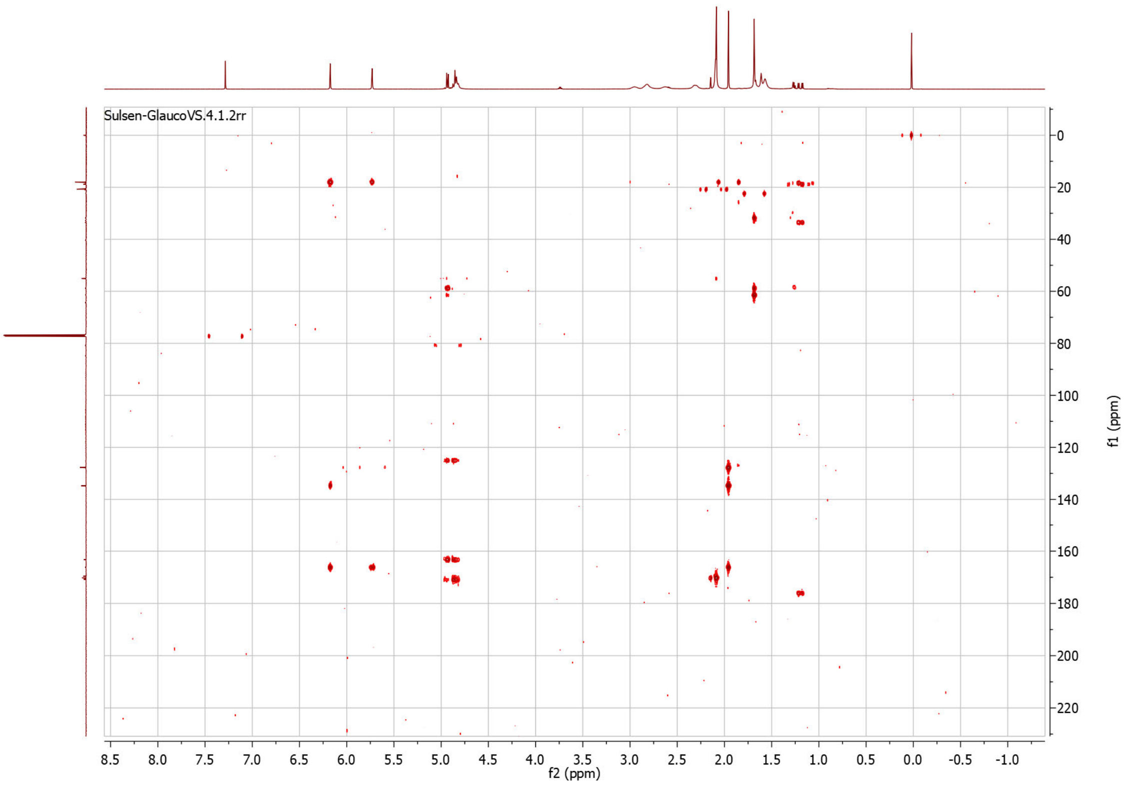

Figure S11. HMBC of compound B (Glaucolide A).
